# Boerhaave syndrome - case report: Síndrome de Boerhaave - relato de caso

**DOI:** 10.1590/1516-3180.2016.0095220616

**Published:** 2016-12-12

**Authors:** Biljana Radovanovic Dinic, Goran Ilic, Snezana Tesic Rajkovic, Tatjana Jevtovic Stoimenov

**Affiliations:** I MD. Associate Professor and Attending Physician, Medical School, University of Niš, and Gastroenterology and Hepatology Clinic, Niš Clinical Center, Niš, Serbia.; II MD. Associate Professor, Medical School, University of Niš, and Institute of Forensic Medicine, Niš, Serbia.; III MD. Attending Physician, Gastroenterology and Hepatology Clinic, Niš Clinical Center, Niš, Serbia.; IV MD. Associate Professor, Medical School, University of Niš, and Institute of Biochemistry, Niš, Serbia.

**Keywords:** Esophagus, Rupture, spontaneous, Hematemesis, Pneumothorax, Emphysema

## Abstract

**CONTEXT:**

Boerhaave syndrome consists of spontaneous longitudinal transmural rupture of the esophagus, usually in its distal part. It generally develops during or after persistent vomiting as a consequence of a sudden increase in intraluminal pressure in the esophagus. It is extremely rare in clinical practice. In 50% of the cases, it is manifested by Mackler's triad: vomiting, lower thoracic pain and subcutaneous emphysema. Hematemesis is an uncommon yet challenging presentation of Boerhaave's syndrome. Compared with ruptures of other parts of the digestive tract, spontaneous rupture is characterized by a higher mortality rate.

**CASE REPORT:**

This paper presents a 64-year-old female patient whose vomit was black four days before examination and became bloody on the day of the examination. Her symptoms included epigastric pain and suffocation. Physical examination showed hypotension, tachycardia, dyspnea and a swollen and painful abdomen. Auscultation showed lateral crackling sounds on inspiration. Ultrasound examination showed a distended stomach filled with fluid. Over 1000 ml of fresh blood was extracted by means of nasogastric suction. Esophagogastroduodenoscopy was discontinued immediately upon entering the proximal esophagus, where a large amount of fresh blood was observed. The patient was sent for emergency abdominal surgery, during which she died. An autopsy established a diagnosis of Boerhaave syndrome and ulceration in the duodenal bulb.

**CONCLUSION:**

Boerhaave syndrome should be considered in all cases with a combination of gastrointestinal symptoms (especially epigastric pain and vomiting) and pulmonary signs and symptoms (especially suffocation).

## RESUMO

**CONTEXTO:**

A síndrome de Boerhaave é uma ruptura longitudinal transmural espontânea do esôfago, normalmente da parte distal. Ela geralmente se desenvolve durante ou após vômitos persistentes como consequência do aumento repentino da pressão intraluminal no esôfago. É extremamente rara na prática clínica. Em 50% dos casos, manifesta-se pela tríade de Mackler: vômitos, dor torácica inferior, enfisema subcutâneo. Hematêmese é uma apresentação incomum porém desafiadora da síndrome de Boerhaave. Em comparação com rupturas de outras partes do tubo digestivo, a ruptura espontânea é caracterizada pela taxa de mortalidade mais elevada.

**RELATO DO CASO:**

O artigo apresenta uma paciente do sexo feminino de 64 anos de idade, cujo vômito era preto, quatro dias antes do exame, e continha sangue no dia do exame. Os sintomas incluíam dor epigástrica e sufocação. No exame físico, foi verificada hipotensão, taquicardia, dispneia e abdômen inchado e doloroso. Ausculta revelou estertores laterais na inspiração. A ultrassonografia mostrou estômago dilatado, preenchido com conteúdo líquido. Sucção nasogástrica evacuou mais de 1.000 ml de sangue fresco. Esofagogastroduodenoscopia foi abortada imediatamente ao se entrar no esôfago proximal, onde foi observada grande quantidade de sangue fresco. A paciente foi encaminhada com urgência para cirurgia abdominal, durante a qual faleceu. Autópsia estabeleceu diagnóstico de síndrome de Boerhaave e úlcera no bulbo-duodenal.

**CONCLUSÃO:**

A síndrome Boerhaave deve ser considerada em todos os casos com uma combinação de sintomas gastrointestinais (especialmente dor epigástrica e vómitos) e sintomas e sinais pulmonares (especialmente sufocação).

## INTRODUCTION

Boerhaave syndrome consists of spontaneous longitudinal transmural rupture of the esophagus. The syndrome is named after a German doctor, Herman Boerhaave, who first described it in 1724.^1^ In comparison with iatrogenic rupture, which may develop during diagnostic or therapeutic endoscopic procedures, traumas or various esophageal diseases, spontaneous rupture most commonly develops during or after persistent vomiting, as a consequence of a sudden increase in intraluminal esophageal pressure. Spontaneous rupture encompasses 15% of all esophageal ruptures.^2^ It is extremely rare in clinical practice. The true incidence of Boerhaave syndrome in the general population is unknown. However, it is considered to be more common than once thought, since many cases of Boerhaave syndrome are only diagnosed postmortem, thus resulting in underreporting and underestimation with regard to both incidence and mortality.^1^^,^^3^ Boerhaave syndrome is seen most frequently among patients aged 50-70 years.^1^

The clinical manifestation of spontaneous rupture of the esophagus depends on the rupture location. In 50% of the cases, it is manifested by Mackler's triad: vomiting, lower thoracic pain and subcutaneous emphysema.^3^^,^^4^

If the diagnosis is not established in time and if appropriate therapeutic measures are not undertaken, serious complications can develop and this may lead to a poor outcome. Compared with ruptures of other parts of the digestive tube, spontaneous rupture of the esophagus has the highest mortality rate.^1^^,^^5^

## CASE REPORT

The patient was a 64-year-old female, with a history of long-term arterial hypertension, who was brought to the Gastroenterology and Hepatology Clinic of the Niš Clinical Center by the emergency medical services. She was admitted presenting with vomiting of fresh blood, black stools, epigastric pain, suffocation and exhaustion.

The problems had first appeared four days before admission in the form of poorly formed black stools and vomiting of small amounts of black substance. She did not see a doctor about these problems. On the day of admission, after vomiting an excessive amount of black substance, she developed a pain in the epigastric region and then began to vomit fresh blood. It was at this stage that she rang the emergency medical services.

Physical examination showed that the patient was alert, adynamic, tachycardiac and easily dyspneic, and her skin was pale. Her blood pressure was 60/40 mmHg. Auscultation of the heart was normal. Auscultation of the lungs showed baseline crackles on inspiration on both sides. The abdomen was tense, especially in the epigastric area and left hypochondrium, with tenderness in the epigastric area. The liver and spleen were of normal size. Appropriate therapy was administered (one ampoule of prantopazole, a total of about 3000 ml of continuous infusion of saline solution and lactated Ringer's solution). The oxygen saturation was 95%. A urinary catheter was placed for monitoring diuresis. An electrocardiogram (ECG) showed sinus tachycardia.

Because of the findings in the abdomen, an ultrasound examination was performed and this showed a distended stomach filled with a large amount of fluid. No free fluid was found in the abdominal cavity. A nasogastric probe was placed in order to extract the contents and perform esophagogastroduodenoscopy (EGD). After inserting the nasogastric probe, about 1,000 ml of fresh blood was extracted. After the hemodynamic status had improved, esophagogastroduodenoscopy was attempted. Immediately upon insertion of the endoscope into the proximal esophagus, reflux of a large amount of fresh blood was observed; further examination was cancelled. The patient was sent for emergency abdominal surgery. However, she died one hour after the first examination.

The laboratory findings and coagulation factors, which were received subsequently, were within normal values. The blood count showed reduced hemoglobin of 70 g/l (reference values: 115-170 g/l) and increased leukocyte count of 12.0 x 10^9^/l (reference values: 4.0-10.0 x 10^9^/l).

**Figure 1:**
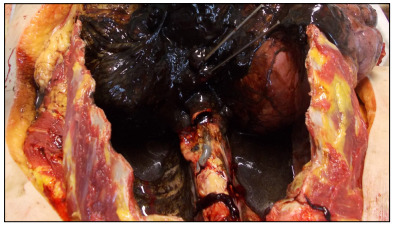
Macroscopic findings from the intrathoracic contents upon opening the thoracic cavity. Note the huge amount of clot.

The autopsy showed 650 ml of dark red to black thick fluid content in the right hemithorax and 600 ml in the left hemithorax (Figure 1). The heart size measurements were 110 x 105 mm. The heart weighed 380 g. The thickness of the cardiac muscle of the left ventricle was 18 mm and of the right ventricle, 6 mm. A rupture along the longitudinal axis was found in the esophagus, in the posterior left section of the esophageal wall, 15 mm from the cardia.

**Figure 2:**
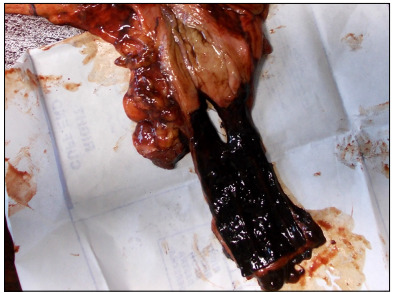
Gross examination of the distal esophagus showing a longitudinal complete rupture 15 mm from the cardia. Note the darkened esophageal mucosa.

The rupture was 30 x 20 mm in size. The esophageal mucosa was smooth and almost completely covered in bloody-black content (Figure 2). There were no foreign bodies in the abdominal cavity. A small amount of blackish liquid was found in the stomach. Numerous small shallow erosions were found in the fundus and body of the stomach.

**Figure 3:**
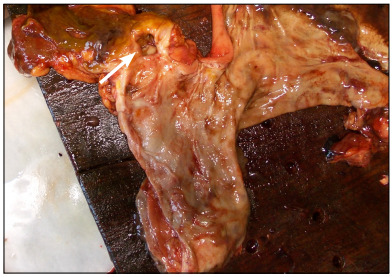
Gross findings from the stomach and duodenum showing deep and wide duodenal ulceration in the duodenal bulb (arrow).

A mucosal injury of depth 13 mm, covering an area of 20 mm x 15 mm with firm borders and blackish background, consistent with a duodenal bulb ulcer, was observed (Figure 3). The walls were firm and vallum-like and the bottom was partially black. Greenish and black content was present throughout the intestines.

Chemical and toxicological analysis on samples of organ tissues, blood and urine did not reveal the presence of any psychoactive substances or pesticides.

The autopsy report declared that the immediate cause of death was hemopneumothorax due to esophageal injury and a chronic duodenal ulcer.

## DISCUSSION

Spontaneous rupture of the esophagus is a rare clinical entity with a high mortality rate.^5^^,^^6^ The pathophysiology of Boerhaave syndrome involves a sudden rise in intraluminal esophageal pressure, thereby forcing the gastric contents against a tight cricopharyngeus muscle.^3^^,^^6^ It most often develops during or after intense vomiting caused by excessive eating or drinking alcohol.^7^ However, spontaneous rupture of the esophagus may occur in the absence of predisposing factors. There are cases of spontaneous esophageal rupture during sleep. In some patients, a muscular layer was missing and this may point to the possibility of anatomical predisposition for the development of rupture.^1^^,^^3^

In the literature, there are cases in which the rupture was also associated with gastroesophageal reflux disease (GERD), Barrett's esophagus, peptic stricture of the esophagus, esophageal dysmotility, paraesophageal hernia or bleeding from a duodenal ulcer, which was the case with our patient.^5^^,^^8^^,^^9^ In our patient, the esophageal rupture was a consequence of excessive vomiting due to the bleeding from the duodenal ulcer.

Spontaneous rupture may occur just above the diaphragm in the posterolateral wall of the esophagus. Perforations are usually longitudinal (0.6-8.9 cm long), with the left side more commonly affected than the right (90%). This is probably due to an anatomical weakness of the left posterolateral aspect of the esophagus just above the diaphragm. Spontaneous rupture is rare below the diaphragm or in the thoracic part of the esophagus.^3^^,^^7^ In our case, the rupture was located in the distal esophagus, 15 mm from the cardia.

The clinical manifestation of Boerhaave syndrome depends on the location of the rupture and the time between its development and examination. Patients with cervical perforation feel pain in the neck and upper half of the thorax. In cases of perforation in the rest of the esophagus, pain is present in the lower part of the thorax and/or upper abdomen. Considering that spontaneous rupture most often happens in the distal esophagus, the majority of patients have Mackler's triad of symptoms and signs: vomiting, lower thoracic pain and subcutaneous emphysema.^3^^,^^4^ However, this triad is rare, which may delay the diagnosis.^10^ In a series of 14 patients with Boerhaave syndrome, only a small percentage had typical signs and symptoms.^3^

The symptoms of Boerhaave syndrome can be nonspecific. Compared with Mallory-Weiss syndrome, Boerhaave syndrome is rarely manifested through hematemesis or other signs of gastrointestinal bleeding, including melena.^1^^,^^3^^,^^6^^,^^10^^,^^11^ In Boerhaave syndrome, the rupture is transmural, which leads to esophageal perforation. In our patient, hematemesis was the chief complaint. To begin with, she was vomiting an excessive amount of black substance as a result of bleeding from ulcers. Excessive vomiting led to spontaneous rupture of the esophagus, which manifested as vomiting of fresh blood.

During physical examination of patients, subcutaneous emphysema is observed in 28%-66% within the first 24 hours. This finding is significant for the initial diagnosis. More typically, subcutaneous emphysema is found later. Besides typical symptoms, atypical symptoms such as hypotension, tachycardia, tachypnea, feverishness and cyanosis may also be present.^1^^,^^7^ Atypical symptoms may be prevented through timely diagnosis. Pneumomediastinum is a significant clinical finding.^10^ Pneumomediastinum is suspected when, during lung auscultation, crunching sounds that are synchronous with the heartbeat are heard (Hamman's sign). This sign is present in around 20% of the cases.^7^

Esophageal rupture may be followed by serious complications, of which the most important ones are mediastinitis and multiple organ dysfunction. Sepsis may develop within a few hours. In such cases, the clinical picture is dominated by signs and symptoms of sepsis, which additionally prevents timely diagnosis and appropriate therapeutic measures.^6^^,^^7^^,^^12^

Laboratory findings are not specific for diagnosing spontaneous esophageal rupture. Serum albumin is normal but may be low, while the globulin fraction may be normal or slightly elevated.^7^ Radiography of the heart and lungs is valuable for the diagnosis. Radiographs usually show signs of pneumomediastinum or pneumothorax or hydropneumothorax if pleural effusion is concurrent.^3^^,^^13^ In cases of perforation of the middle third of the esophagus, pleural effusion is present on the right side, while in cases of rupture of the distal esophagus, pleural effusion is present on the left side.^5^ Diagnostic thoracentesis shows the presence of food remnants, increased amylase and pH below 6. The presence of pneumomediastinum with data including vomiting and chest pain are almost definite signs of Boerhaave syndrome. Overall, 10% of chest radiographs are normal.^7^^,^^14^

Esophagography is an important imaging examination for confirming the diagnosis and the location of perforation because it shows extravasation of contrast into the pleural space. The procedure is performed with water-soluble contrast, such as Gastrografin, since barium may cause severe mediastinitis. Esophagography with Gastrografin is 90% sensitive.^7^

Thoracic computed tomography imaging is indicated for making the diagnosis in patients who do not tolerate esophagography. During the procedure, localized fluid collection is observed, as well as periesophageal air collection.^1^^,^^15^^,^^16^ The role of EGD in the early diagnostic work-up of patients with suspected esophageal perforation has been disputed.^17^ EGD is not recommended for diagnosing Boerhaave syndrome, since it may increase the rupture and the amount of air in the mediastinum and pleural space.^13^ In cases with hematemesis, such as in our patient, the procedure was attempted in order to ascertain the source of bleeding.

The treatment for Boerhaave syndrome is both conservative and surgical. The goals of pharmacotherapy are to reduce morbidity and to prevent complications. Surgical management is generally required for both spontaneous rupture and traumatic perforation.^14^^,^^18^ Endoscopic stent insertion offers a promising alternative. The mortality rate varies depending on the time that has elapsed since development of the rupture and its recognition and treatment. If treatment is not started within 24 hours from the onset of symptoms, the mortality rate is 25%; after 24 hours, it is 65%; and after 48 hours, it is 75%-89%.^19^

We reviewed the literature in Medline, PubMed, Embase and Lilacs using the English keywords "Esophagus", "Rupture, spontaneous", "Hematemesis" and "Pneumothorax"; and the Portuguese words "Esôfago", "Ruptura espontânea", "Hematêmese" and "Pneumotórax" (Table 1).

**Table 1:**
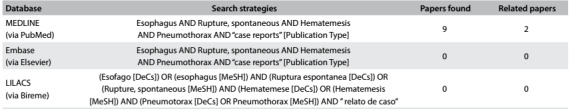
Literature search in medical databases for case reports on Boerhaave syndrome. The literature search was conducted on May 4, 2016

## CONCLUSION

Boerhaave syndrome should be considered in all patients with a combination of gastrointestinal symptoms (epigastric pain and vomiting) and pulmonary symptoms (suffocation), even when all the signs and symptoms (lower thoracic pain and subcutaneous emphysema) of this disease are absent. Early clinical suspicion will lead to timely diagnosis and maximize the survival chances for the patient.

## References

[1] Dellon ES, Shaheen NJ, Yamada T (2009). Miscellaneous diseases of the esophagus: foreign bodies, physical injury and systemic and dermatological diseases. Textbook of Gastroenterology.

[2] Brinster CJ, Singhal S, Lee L (2004). Evolving options in the management of esophageal perforation. Ann Thorac Surg.

[3] Garas G, Zarogoulidis P, Efthymiou A (2014). Spontaneous esophageal rupture as the underlying cause of pneumothorax early recognition is crucial. J Thorac Dis.

[4] Venø S, Eckardt J (2013). Boerhaave's syndrome and tension pneumothorax secondary to Norovirus induced forceful emesis. J Thorac Dis.

[5] Reardon ES, Martin LW (2015). Boerhaave's syndrome presenting as a mid-esophageal perforation associated with a right-sided pleural effusion. J Surg Case Rep.

[6] de Schipper JP, Pull ter Gunne AF, Oostvogel HJ, van Laarhoven CJ (2009). Spontaneous rupture of the oesophagus Boerhaave's syndrome in 2008. Literature review and treatment algorithm. Dig Surg.

[7] Roy PK, Murphy ME, Kalapatapu V, Bashir S, Mujibur R Boerhaave Syndrome. Medscape.

[8] Tsalis K, Vasiliadis K, Tsachalis T (2008). Management of Boerhaave's syndrome report of three cases. J Gastrointestin Liver Dis.

[9] Yang ST, Devanand A, Tan KL, Eng PC (2003). Boerhaave's syndrome presenting as a right-sided pleural effusion. Ann Acad Med Singapore.

[10] Fikfav V, Gaur P, Kim MP (2014). Endoscopic management of Boerhaave's syndrome presenting with hematemesis. J Surg Case Rep.

[11] Søreide JA, Viste A (2011). Esophageal perforation diagnostic work-up and clinical decision-making in the first 24 hours. Scand J Trauma Resusc Emerg Med.

[12] Woo KM, Schneider JI (2009). High-risk chief complaints I: chest pain--the big three. Emerg Med Clin North Am.

[13] Eckstein M, Sean O, Marx J A (2014). Henderson. Thoracic trauma, esophagus perforation. Rosen's Emergency Medicine. Concepts and Clinical Practice.

[14] Kollmar O, Lindemann W, Richter S (2003). Boerhaave's syndrome primary repair vs. esophageal resection--case reports and meta-analysis of the literature. J Gastrointest Surg.

[15] Duman H, Bakirci EM, Karadag Z, Ugurlu Y (2014). Esophageal rupture complicated by acute pericarditis. Turk Kardiyol Dern Ars.

[16] Vial CM, Whyte RI (2005). Boerhaave's syndrome: diagnosis and treatment. Surg Clin North Am.

[17] Arantes V, Campolina C, Valerio SH (2009). Flexible esophagoscopy as a diagnostic tool for traumatic esophageal injuries. J Trauma.

[18] Huber-Lang M, Henne-Bruns D, Schmitz B, Wuerl P (2006). Esophageal perforation principles of diagnosis and surgical management. Surg Today.

[19] Schweigert M, Beattie R, Solymosi N (2013). Endoscopic stent insertion versus primary operative management for spontaneous rupture of the esophagus (Boerhaave syndrome) an international study comparing the outcome. Am Surg.

